# Hyperreflective retinal foci are associated with retinal degeneration after optic neuritis in neuromyelitis optica spectrum disorders and multiple sclerosis

**DOI:** 10.1111/ene.70038

**Published:** 2025-01-10

**Authors:** Philipp Klyscz, Ifat Vigiser, Gilberto Solorza Buenrostro, Seyedamirhosein Motamedi, Carla Johanna Leutloff, Patrick Schindler, Tanja Schmitz‐Hübsch, Friedemann Paul, Hanna Gwendolyn Zimmermann, Frederike Cosima Oertel

**Affiliations:** ^1^ Experimental and Clinical Research Center Max Delbrück Center for Molecular Medicine Berlin and Charité—Universitätsmedizin Berlin, Corporate Member of Freie Universität Berlin and Humboldt‐Universität zu Berlin Berlin Germany; ^2^ Department of Neurology Charité—Universitätsmedizin Berlin, Corporate Member of Freie Universität Berlin and Humboldt‐Universität zu Berlin Berlin Germany; ^3^ Neuroscience Clinical Research Center (NCRC) Charité—Universitätsmedizin Berlin, Corporate Member of Freie Universität Berlin and Humboldt‐Universität zu Berlin Berlin Germany; ^4^ Neuroimmunology and Multiple Sclerosis Unit, Neurology Institute Tel Aviv Sourasky Medical Center Tel Aviv Israel; ^5^ Sackler Faculty of Medicine Tel Aviv University Tel Aviv Israel; ^6^ Einstein Center Digital Future Berlin Germany

**Keywords:** hyperreflective retinal foci, multiple sclerosis, neuromyelitis optica spectrum disorder, OCT

## Abstract

**Background:**

Hyperreflective retinal foci (HRF) visualized by optical coherence tomography (OCT) potentially represent clusters of microglia. We compared HRF frequencies and their association with retinal neurodegeneration between people with clinically isolated syndrome (pwCIS), multiple sclerosis (pwMS), aquaporin 4‐IgG positive neuromyelitis optica spectrum disorder (pwNMOSD), and healthy controls (HC)—as well as between eyes with (ON^+^eyes) and without a history of optic neuritis (ON^−^eyes).

**Methods:**

Cross‐sectional data of pwCIS, pwMS, and pwNMOSD with previous ON and HC were acquired at Charité—Universitätsmedizin Berlin. HRF analysis was performed manually on the central macular OCT scan. Semi‐manual OCT segmentation was performed to acquire the combined ganglion cell and inner plexiform layer (GCIPL), inner nuclear layer (INL), and peripapillary retinal nerve fiber layer (pRNFL) thickness. Group comparisons were performed by linear mixed models.

**Results:**

In total, 227 eyes from 88 patients (21 pwCIS, 32 pwMS, and 35 pwNMOSD) and 35 HCs were included. HRF in GCIPL and INL were more frequently detected in pwCIS, pwMS, and pwNMOSD than HCs (*p* < 0.001 for all comparisons) with pwCIS exhibiting the greatest numbers. ON^+^eyes of pwMS had less HRF in GCIPL than ON^−^eyes (*p* = 0.036), but no difference was seen in pwCIS and pwNMOSD. HRF GCIPL were correlated to GCIPL thickness in ON^+^eyes in pwMS (*p* = 0.040) and pwNMOSD (*p* = 0.031).

**Conclusion:**

HRF occur in ON^+^eyes and ON^−^eyes across neuroinflammatory diseases. In pwMS and pwNMOSD, HRF frequency was positively associated with GCIPL thickness indicating that HRF formation might be dependent on retinal ganglion cells.

## INTRODUCTION

Multiple sclerosis (MS) and neuromyelitis optica spectrum disorders (NMOSD) are autoimmune inflammatory diseases of the central nervous system (CNS) characterized by recurrent inflammation, demyelination, and neurodegeneration [1, 2]. MS is one of the most common neuroinflammatory diseases affecting more than 2.8 million people globally [3]. The majority of people with MS (pwMS) exhibit a relapsing disease course, which is defined by recurrent attacks [1]. If a patient has a first attack but does not fulfill the diagnostic criteria for MS, it is considered a clinically isolated syndrome (CIS) [4]. Up to 70% of people with CIS (pwCIS) will subsequently be diagnosed with MS. [5] In contrast to MS, NMOSD is a rare disease with a prevalence of 4–13/100,000 people [6]. Most people with NMOSD (pwNMOSD) have antibodies against aquaporin‐4 (AQP4‐IgG), an astrocytic water channel, suggesting an astrocytopathy as part of the disease pathology [7]. Both diseases share several clinical characteristics including the common manifestation of optic neuritis (ON). ON describes an inflammation of the optic nerves that subsequently leads to demyelination, neurodegeneration, and severe vision loss [8].

Microglia, the resident immune cells of the CNS, are key players in MS and NMOSD pathology. During neuroinflammation, microglia recruit lymphocytes, trigger T‐cell activation, and release inflammatory cytokines [9]. Yet, microglia are also involved in maintaining CNS homeostasis and removing apoptotic cells [9]. In MS, widespread activation of microglia in the CNS including on the rim of chronic active lesions is observed [10]. Thus, they are often considered drivers of chronic disease progression in MS. [10] In NMOSD, microglial activation was also shown in early pathology, but it remains unclear to which extent they fuel ongoing disease activity [11]. In murine NMOSD models, microglia seem to be necessary for NMOSD pathogenesis [12]. Despite the distinct pathology of MS and NMOSD, microglia might thus be promising therapeutic targets. Nevertheless, quantifying microglia and microglial activation in vivo remains challenging. Imaging modalities such as 18‐kDa translocator protein (TSPO) positron emission tomography (PET) have been proposed for the analysis of microglia. Yet, TSPO‐PET is still not widely available and poses an additional radiation burden [13]. Non‐invasive imaging techniques for the quantification of microglia and microglial activation are therefore highly warranted.

A potential alternative for measuring microglial activity might be retinal optical coherence tomography (OCT). OCT is currently used in clinical practice to non‐invasively measure retinal structural parameters such as peripapillary retinal nerve fiber layer (pRNFL), combined ganglion cell and inner plexiform layer (GCIPL), or inner nuclear layer (INL) — particularly for the diagnosis of acute and past ON [14, 15, 16]. Several studies have validated pRNFL and GCIPL as markers of retinal neurodegeneration as well as mirroring brain atrophy and predicting future disease activity [17, 18]. INL was suggested as a marker of inflammatory disease activity and therapy response [15]. To quantify microglia and microglial activity, the novel OCT parameter hyperreflective retinal foci (HRF) have become of increasing interest. HRF are present in healthy people in various degrees but elevated numbers of HRF have been described in several ophthalmologic and neurological diseases including diabetic retinopathy [19], Fabry disease [20], and age‐related macular degeneration (AMD) [21] as well as recently in MS. [22] Recent pathology data of HRF in AMD indicate HRF in the outer retina to mainly consist of melanolipofuscin‐laden mononuclear phagocytes and differentiated microglia [23]. In a mice model of diabetic retinopathy, an increased microglial activity was also detected in the area of HRF [24]. Serological evidence of elevated proinflammatory cytokines in the cerebrospinal fluid of pwMS in relation to HRF numbers in the inner retina indicates them also as a marker of activated microglia in MS. [21, 25] Although promising, the definitive etiology of HRF has not been proven.

Previous research on HRF in CNS inflammatory diseases focused solely on pwMS without a clinical history of ON. To expand the clinical usability of HRF, we here 1) compared the frequency of HRF between eyes of pwCIS, pwMS, and pwNMOSD with previous ON (ON^+^eyes) and without a history of ON (ON^−^eyes) and 2) investigated the association of HRF with markers of retinal neurodegeneration.

## METHODS

### Study population

Data for this retrospective analysis were derived from three completed cohort studies between 2011 and 2021 at the Neuroscience Clinical Research Center (NCRC) at Charité—Universitätsmedizin Berlin, Germany. Adult subjects with CIS or early MS that experienced an ON as a first clinical manifestation were included from the CIS‐cohort (EA1/182/10). Inclusion criteria consisted of diagnosis of CIS within six months of study inclusion or diagnosis of MS within two years according to the 2017 McDonald criteria [4]. Exclusion criteria were a diagnosis of secondary progressive MS and any ophthalmologic disease that might interfere with OCT. Subjects with a minimum age of 18 years, a diagnosis of AQP4‐IgG‐positive NMOSD according to the 2015 International Consensus diagnostic criteria for NMOSD and a medical history of ON were included from the NMO‐cohort study (EA1/041/14). AQP4‐IgG seropositivity was tested by fixed cell‐based assay (Euroimmun, Lübeck, Germany). Healthy controls (HC) were enrolled from the VIMS study (EA1/163/12) and were free of any ophthalmologic or neurologic disease. HCs were age‐ and sex‐matched to enrolled subjects with NMOSD. Collected characteristics included sex, age, expanded disability severity scale (EDSS) score, disease duration, number of previous ON, and disease‐modifying therapies (DMT). In accordance with Wiendl et al. [26] low‐efficacy DMT consisted of interferon, glatiramer acetate, dimethyl fumarates, azathioprine, and mycophenolate mofetil. High‐efficacy treatment consisted of rituximab, tocilizumab, and belimumab.

### Optical coherence tomography

All retinal OCT examinations were performed using Heidelberg Engineering Spectralis spectral domain OCT (Heidelberg Engineering, Heidelberg, Germany) with automatic real‐time (ART) image averaging without pupillary dilatation and were quality assessed according to OSCAR‐IB criteria [27, 28]. The pRNFL thickness was calculated with activated eye tracker using 3.4‐mm ring scans around the optic nerve head (12°, 1536 A‐scans 16 ≤ ART ≤100). The GCIPL and INL thickness was measured in a 6‐mm diameter around the fovea based on a macular volume scan (25° × 30°, 61 B‐scans with 768 A‐scans per B‐scan, ART = 15). Segmentation of pRNFL and GCIPL was performed semi‐automatically using Heidelberg Eye Explorer (Eye Explorer 1.9.10.0 with viewing module 6.0.9.0; Heidelberg Engineering) and corrected, if necessary, by an experienced rater (CL). Reported results adhered to the APOSTEL 2.0 recommendations [29, 30]. OCT images of study participants were blinded regarding ON status but not for different disease groups.

### Hyperreflective retinal foci

**FIGURE 1:**
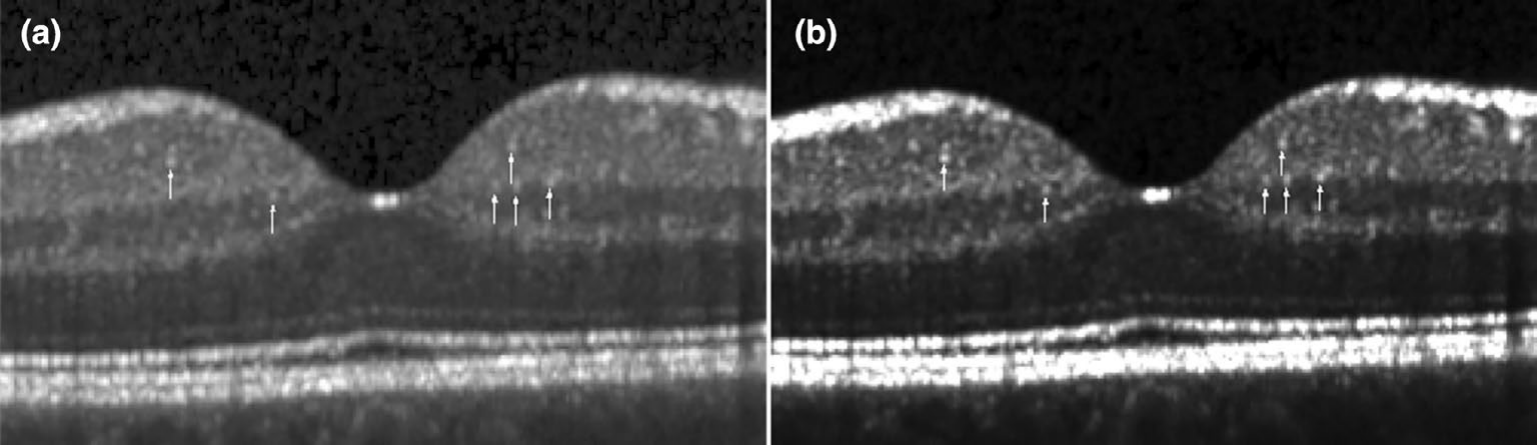
Macular optical coherence tomography scans in a patient with multiple sclerosis. Hyperreflective retinal foci are marked with white arrows. (a) shows the original scan and (b) the adjusted scan after image processing (own depiction).

Counting of HRF were performed using the central B‐scan crossing the fovea in a 3000 μm diameter around the center by an experienced rater (IV). As previously described, HRF were defined as small (< 30 μm), punctiform elements with a reflectivity similar to the RNFL but without any back shadowing [31]. HRF were quantified in the GCIPL and INL separately. HRF quantification was confirmed for all scans by a second independent rater (PK). For analyses of HRF, we used the open‐source software OCT‐marker (OCT‐marker, Kay Gawlik, Charité—Universitätsmedizin Berlin, Germany) that enables image modification (i.e., gamma correction, contrast, brightness) to improve visibility of HRF and reduction of background noise (Figure 1). The interrater reliability between the two independent raters (IV, PK) was assessed using the intraclass correlation coefficient (ICC). The ICC for quantification of HRF was 0.8 and therefore classified as good. In cases of quantification differences between the two raters, an expert consensus between the two raters was reached during the secondary review.

### Statistical methods

All statistical analysis and graphical visualizations were performed using R (version 4.2.2). *p*‐values <0.05 were defined as statistically significant. *p*‐values <0.07 were defined as statistical trend. Categorical variables are described in absolute numbers and relative percentages, ordinal variables are reported as median and interquartile range (IQR), and continuous variables are presented as mean and standard deviation (SD). Total HRF consisted of the sum of HRF in the GCIPL and INL. Linear mixed models were employed for all analyses regarding pRNFL, GCIPL, and INL thickness and HRF numbers to account for multiple observations in the same patient (left and right eye). First, differences between the disease entities were examined. Second, a comparison of eyes with and without ON within the respective disease group was performed. β and standard errors were reported. Linear mixed models were not applicable when analyzing a potential association between HRF and retinal structural parameters distinctly in ON^−^eyes and ON^+^eyes of pwCIS and pwMS as these patients only had a single unilateral ON. In these cases, spearman correlations were applied, and correlation coefficients (*r*) were reported. Due to the exploratory nature of this study, the uncorrected results are being reported. A preliminary adjustment using false discovery rate correction revealed only an impact on associations between HRF numbers and retinal structural parameters in ON^+^eyes (Tables S1–S3).

### Ethics approval and consent to participate

All cohort studies were approved by the local ethics committee at Charité—Universitätsmedizin Berlin (EA1/182/10, EA1/041/14 EA1/163/12). The studies were conducted in accordance with the Declaration of Helsinki in its currently applicable version and the applicable European and German laws. All participants provided written informed consent.

## RESULTS

### Study population

In total, 227 eyes of 123 people, which included 35 (28.5%) HC, 35 (28.5%) pwNMOSD, 32 (26.0%) pwMS, and 21 (17.0%) pwCIS (Table 1). Nineteen eyes were excluded due to inadequate macular OCT image quality. Study population characteristics are summarized in Table 1. PwCIS and pwMS were significantly younger than HC and NMOSD (pwCIS: HC *p* < 0.001, pwNMOSD *p* < 0.001; pwMS: HC *p* < 0.001, pwNMOSD *p* < 0.001). Further, pwNMOSD were significantly more often female than pwCIS (*p* = 0.049) and pwMS. No differences were seen regarding age and sex between pwCIS and pwMS as well as between pwNMOSD and HC.

**TABLE 1:** Characteristics of the study population.

Characteristic	pwCIS	pwMS	pwNMOSD	HC
	*n* = 21	*n* = 32	*n* = 35	*n* = 35
Number of eyes (*n*)	39	60	60	68
Sex, female (*n* [%])	15 (71)	21 (66)	33 (94)	28 (88)
Age in years (mean [SD])	36 (9)	32 (8)	48 (14)	49 (11)
EDSS (median [IQR])	1.5 (1.0–2.0)	1.5 (1.0–1.5)	3.5 (2.0–4.0)	.
Disease duration in months (mean [SD])	5 (2)	6 (5)	65 (62)	.
Eyes with a history of ON (*n* [%])	18 (46)	30 (50)	23 (38)	.
Time since last ON in months (mean [SD])	5 (2)	5 (4)	47 (43)	.
Number of ON episodes (median [IQR])	1 (1–1)	1 (1–1)	2 (1–2)	.
High‐efficacy DMT (*n* [%])	0	0	9 (26)	.
Low‐efficacy DMT (*n* [%])	3 (14)	16 (50)	21 (60)	.

*Note:* Low‐efficacy DMT: interferon, glatiramer acetate, dimethyl fumarates, azathioprine, and mycophenolate mofetil. High‐efficacy DMT: rituximab, tocilizumab, and belimumab. “.” indicate not applicable to healthy controls (HC).

Abbreviations: DMT, disease‐modifying therapy; EDSS, expanded disability status scale; HC, healthy control; IQR, interquartile range; ON, optic neuritis; pwCIS, people with the clinically isolated syndrome; pwMS, people with multiple sclerosis; pwNMOSD, people with neuromyelitis optica spectrum disorder; SD, standard deviation.

### HRF are a retinal feature in NMOSD, CIS, and MS

Total HRF were not associated with age (*β* = −0.02 ± 0.05 [n.s.]) or sex in the whole cohort (*β* = −0.66 ± 1.47 [n.s.]) as well as in all subgroups (data not shown). Longer disease duration was associated with less occurrence of total HRF in pwNMOSD (*β* = −0.07 ± 0.02 [*p* < 0.001]) but not in pwsCIS (*β* = 0.81 ± 0.48 [n.s.]) or pwMS (*β* = −0.03 ± 0.19 [n.s.]). Yet, a longer time interval from the last ON showed a trend of less total HRF (*β* = −0.05 ± 0.03 [*p* = 0.064]). When comparing the influence of DMT in pwCIS and pwMS on HRF frequency, patients taking DMT had fewer HRF in GCIPL, even though this was not a significant difference (*β* = −1.47 ± 1.06 [n.s.]).

**FIGURE 2:**
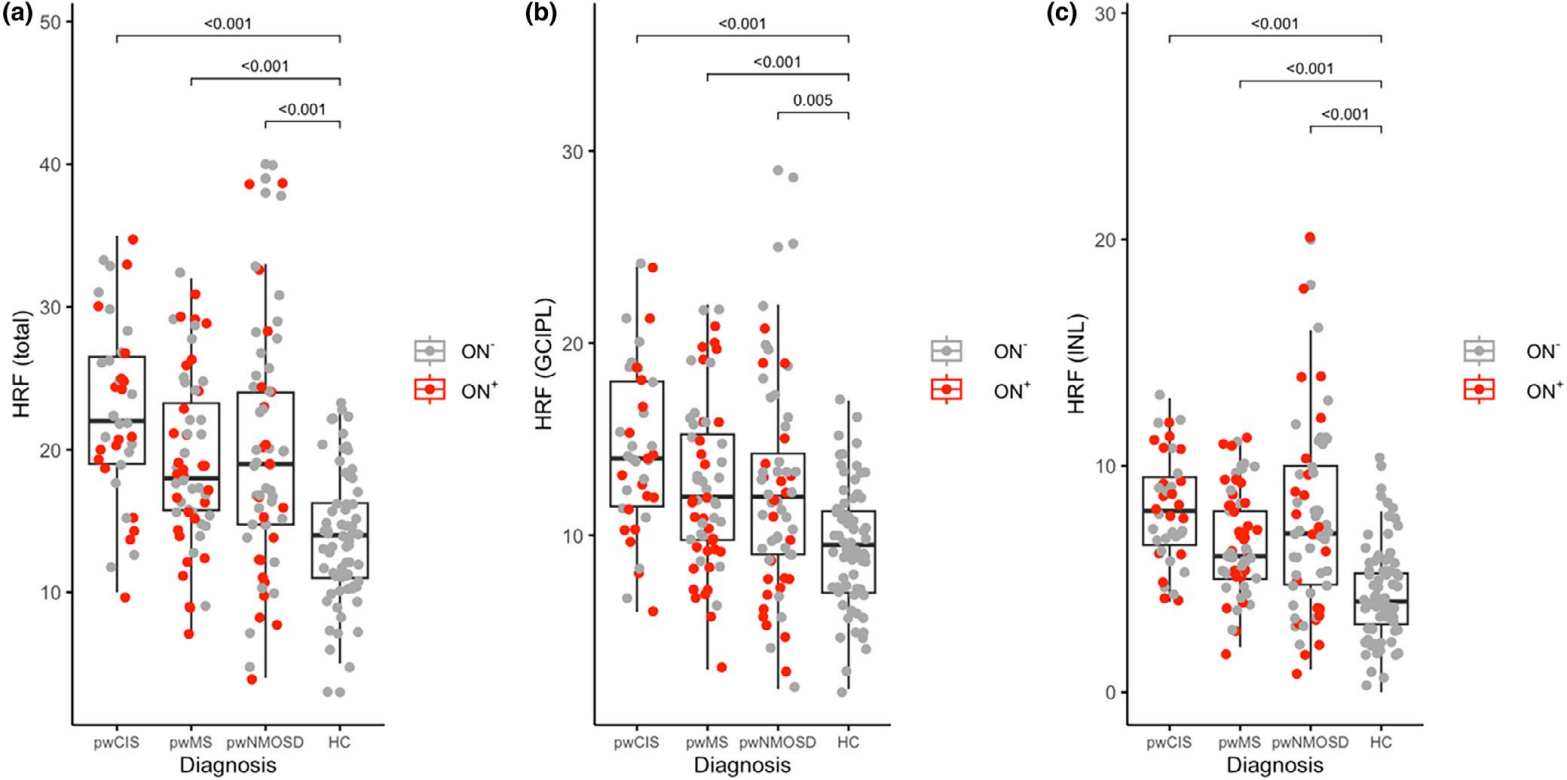
Boxplots comparing numbers of HRF for each disease with HC. Depicted are total HRF (a), HRF in GCIPL (b), and HRF in INL (c) for pwCIS (left), pwMS (second from left), pwNMOSD (third from left), and HC (right). Total HRF are the sum of GCIPL and INL HRF. Pairwise comparisons were performed using linear mixed models between all eyes of pwCIS, pwMS, and pwNMOSD and HC. GCIPL, combined ganglion cell and inner plexiform layer; HC, healthy controls; HRF, hyperreflective retinal foci; INL, inner nuclear layer; ON ^+^, eyes with previous optic neuritis; ON ^−^, eyes without previous optic neuritis; pwCIS, people with clinically isolated syndrome; pwMS, people with multiple sclerosis; pwNMOSD, people with neuromyelitis optica spectrum disorder.

Total HRF counts were higher in pwCIS, pwMS, and pwNMOSD compared to HC (pwNMOSD *β* = −5.69 ± 1.39 [*p* < 0.001], pwCIS *β* = −8.83 ± 1.09 [*p* < 0.001], pwMS *β* = −5.44 ± 1.07 [*p* < 0.001], Figure 2). These differences persisted when comparing HRF in GCIPL (pwNMOSD *β* = −2.55 ± 0.87 [*p* = 0.005], pwCIS *β* = −5.01 ± 0.74 [*p* < 0.001], pwMS *β* = −3.07 ± 0.76 [*p* < 0.001]) and INL separately (pwNMOSD *β* = −3.15 ± 0.68 [*p* < 0.001], pwCIS *β* = −3.80 ± 0.49 [*p* < 0.001], pwMS *β* = −2.36 ± 0.43 [*p* < 0.001]).

PwCIS had higher HRF counts in GCIPL (*β* = −2.06 ± 1.02 [*p* = 0.048]) and INL than pwMS (*β* = −1.43 ± 0.57 [*p* = 0.015]). PwCIS also had higher HRF counts in GCIPL (*β* = −2.58 ± 1.17 [*p* = 0.032]) but not in INL compared to pwNMOSD (*β* = −0.67 ± 0.89 [n.s.]). No significant differences in HRF count were seen between pwMS and pwNMOSD.

### HRF frequency is associated with ON history in pwMS but not in pwCIS or pwNMOSD

**FIGURE 3:**
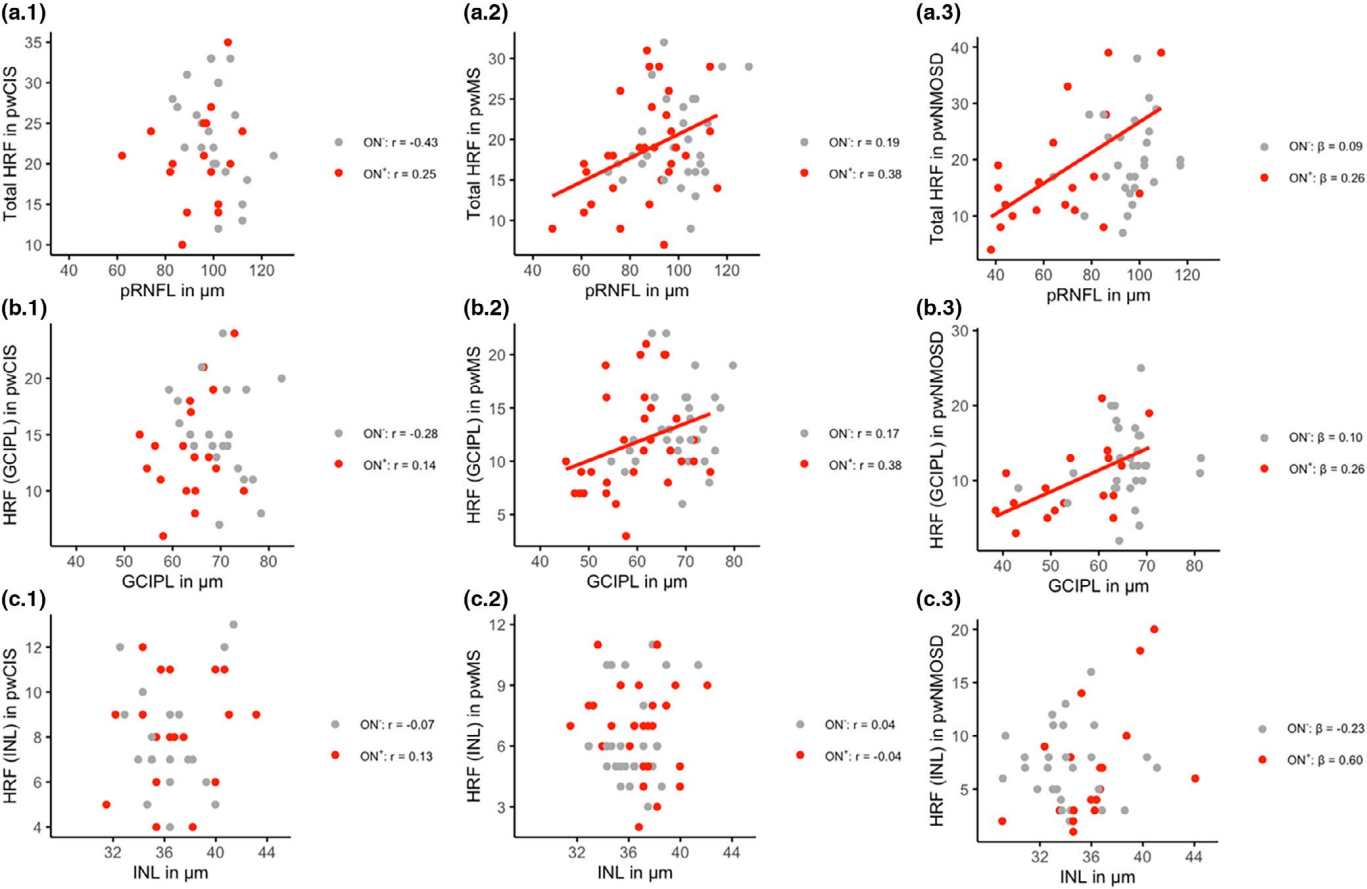
Scatterplots depicting the association between number of HRF and retinal structural parameters for each disease. (a) for Total HRF (sum of HRF in GCIPL and INL) and pRNFL thickness, (b) for HRF in GCIPL and GCIPL thickness, and (c) for HRF in INL and INL thickness. The first column shows scatterplots for pwCIS (a.1, b.1, c.1), the second column for pwMS (a.2, b.2, c.2), and the third column for pwNMOSD (a.3, b.3, c.3). Depicted lines indicate a significant linear association separated for ON^+^eyes and ON^−^eyes. GCIPL, combined ganglion cell and inner plexiform layer; HRF, hyperreflective retinal foci; INL, inner nuclear layer; ON ^−^eyes, eyes without previous ON; ON ^+^eyes, eyes with previous ON; pRNFL, peripapillary retinal nerve fiber layer; pwCIS, people with clinically isolated syndrome; pwMS, people with multiple sclerosis; pwNMOSD, people with neuromyelitis optica spectrum disorder.

ON^+^eyes of pwMS had a lower number of HRF in the GCIPL but not in the INL (Figure 3) than ON^−^eyes (HRF GCIPL: *β* = −1.71 ± 0.78 [*p* = 0.036]; HRF INL: *β* = −0.45 ± 0.42 [*p* = n.s.]). In pwCIS and pwNMOSD, HRF number did not differ between ON^+^eyes and ON^−^eyes in the GCIPL (pwCIS: *β* = −1.47 ± 1.42 [n.s.]; pwNMOSD: *β* = −2.05 ± 1.33 [n.s.]) nor in the INL (pwCIS: *β* = 0.28 ± 0.61 [n.s.]; pwNMOSD: *β* = 0.41 ± 0.87 [n.s.]; Table 2). When comparing ON^+^eyes between different etiologies, pwCIS had more HRF in the GCIPL but not in INL than pwNMOSD (HRF GCIPL: *β* = −3.28 ± 1.52 [*p* = 0.038]; HRF INL: *β* = −0.86 ± 1.30 [n.s.]). HRF frequencies did not differ significantly in ON^+^eyes between other groups. Across diagnoses, a greater time interval from last ON showed a trend of less total HRF and HRF in GCIPL (Total HRF *β* = −0.05 ± 0.03 [*p* = 0.064]; HRF GCIPL *β* = −0.04 ± 0.02 [*p* = 0.056]). Adjusting for GCIPL thickness did not influence this trend (data not shown).

**TABLE 2:** Retinal structural parameters and number of HRF for each disease separated by ON status. Statistical analyses were performed using linear mixed models and are displayed for the ON^−^eyes versus ON^+^eyes comparison within each diagnostic subgroup.

	pwCIS				pwMS				pwNMOSD			
	*n* = 21				*n* = 32				*n* = 35			
	ON^−^eye	ON^+^eye	*B* ± SE	*p*	ON^−^eye	ON^+^eye	*B* ± SE	*p*	ON^−^eye	ON^+^eye	B ± SE	*p*
pRNFL in μm (mean [SD])	101 (11)	94 (13)	−6.9 ± 2.2	0.006	98 (14)	86 (17)	−12.4 ± 2.9	<0.001	94 (13)	64 (20)	−26.2 ± 4.6	<0.001
GCIPL in μm (mean [SD])	70 (6)	64 (6)	−6.6 ± 1.2	<0.001	68 (6)	60 (8)	−8.9 ± 1.5	<0.001	64 (9)	53 (10)	−8.1 ± 1.9	<0.001
INL in μm (mean [SD])	36 (3)	37 (3)	0.5 ± 0.4	0.155	36 (2)	37 (2)	0.7 ± 0.2	0.005	34 (3)	36 (4)	0.5 ± 0.3	0.069
Total HRF (median, IQR)	22 (19–27)	21 (19–25)	−1.1 ± 1.9	0.565	18 (16–23)	18 (14–23)	−1.3 ± 1.0	0.217	19 (17–26)	16 (11–22)	−1.5 ± 2.0	0.464
HRF GCIPL (median, IQR)	14 (12–17)	13 (10–17)	−1.5 ± 1.4	0.309	12 (11–16)	10.5 (8–15)	−1.7 ± 0.8	0.036	12 (10–17)	10 (7–13)	−2.0 ± 1.3	0.131
HRF INL (median, IQR)	7 (6–10)	8.5 (7–11)	0.3 ± 0.6	0.656	6 (5–8)	7 (5–8)	0.4 ± 0.4	0.294	7 (5–9)	7 (4–10)	0.4 ± 0.9	0.639

Abbreviations: GCIPL, combined ganglion cell and inner plexiform layer; HRF, hyperreflective retinal foci; INL, inner nuclear layer; IQR, interquartile range; ON^−^eyes, eyes without previous ON; ON^+^eyes, eyes with previous ON; pRNFL, peripapillary retinal nerve fiber layer; pwCIS, people with clinically isolated syndrome; pwMS, people with multiple sclerosis; pwNMOSD, people with neuromyelitis optica spectrum disorder; SD, standard deviation.

In ON^−^eyes, HRF in the GCIPL and INL were more frequently detected in patients' eyes than in HC eyes (HRF GCIPL: pwNMOSD *β* = −3.47 ± 0.90 [*p* < 0.001], pwCIS *β* = −5.69 ± 0.86 [*p* < 0.001], pwMS *β* = −3.77 ± 0.74 [*p* < 0.001]; HRF INL: pwNMOSD *β* = −2.92 ± 0.58 [*p* < 0.001], pwCIS *β* = −3.67 ± 0.54 [*p* < 0.001], pwMS *β* = −2.13 ± 0.47 [*p* < 0.001]). Further, ON^−^eyes of pwCIS had significantly higher numbers of HRF in INL but not in GCIPL compared to ON^−^eyes of pwMS (HRF GCIPL: *t* = −1.66 [*p* = 0.105]; HRF INL: *t* = −2.26 [*p* = 0.029]). A pairwise comparison of ON^−^eyes between diseases did not yield any significant associations.

### Correlation between HRF and retinal structural parameters

Across subjects with preexisting demyelinating disease, ON^+^eyes had a thinner pRNFL and GCIPL compared to ON^−^eyes (pRNFL: pwMS *β* = −12.40 ± 2.95 [*p* < 0.001], pwCIS: *β* = −6.88 ± 2.20 [*p* = 0.005], pwNMOSD: *β* = −26.21 ± 4.55 [*p* < 0.001]; GCIPL: pwMS *β* = −8.89 ± 1.47 [*p* < 0.001], pwCIS: *β* = −6.61 ± 1.24 [*p* < 0.001], pwNMOSD: *β* = −8.07 ± 1.90 [*p* < 0.001]).

In pwMS and pwNMOSD, Total HRF were positively associated to pRNFL (pwMS: *β* = 0.11 ± 0.04 [*p* = 0.012]; pwNMOSD *β* = 0.16 ± 0.05 [*p* = 0.004]) and GCIPL thickness (pwMS: *β* = 0.17 ± 0.07 [*p* = 0.026]; pwNMOSD *β* = 0.35 ± 0.13 [*p* = 0.010]) but not in pwCIS (pRNFL: *β* = −0.01 ± 0.09 [n.s.]; GCIPL: *β* = 0.06 ± 0.16 (n.s.)). Furthermore, in pwMS and pwNMOSD number of HRF in GCIPL were positively associated to GCIPL thickness (pwMS: *β* = 0.15 ± 0.06 [*p* = 0.015]; pwNMOSD: *β* = 0.22 ± 0.08 [*p* = 0.006]). These associations persisted in ON^+^eyes of pwMS and pwNMOSD but not in ON^−^eyes (ON^+^eyes: pwMS *r* = 0.38 [*p* = 0.040]; pwNMOSD *β* = 0.26 ± 0.11 [*p* = 0.031]; ON^−^eyes: pwMS *r* = 0.17 [n.s.]; pwNMOSD *β* = 0.10 ± 0.14 [n.s.]). There was no significant association for a number of Total HRF or HRF in INL with INL thickness (Figure 3).

## DISCUSSION

This study shows higher numbers of HRF in the GCIPL and INL in pwCIS, pwMS, and pwNMOSD than in HC with pwCIS presenting with the highest HRF numbers. Further, in pwMS and pwNMOSD, HRF in GCIPL were positively associated with GCIPL thickness in ON^+^eyes. This confirms and extends prior cross‐sectional and longitudinal data comparing HRF frequencies in pwMS and HC [22, 32].

Microglia play an active role during acute inflammation and phagocytosis of damaged tissue during the post‐acute stage. We and others showed in animal models of neuroinflammation such as experimental autoimmune encephalomyelitis (EAE), that the number of retinal microglia increases specifically during these phases and decreases thereafter [33]. A prior experimental study also suggested that HRF might be a surrogate marker of localized blood‐retina barrier (BRB) disruption by activated microglia [24]. This is in line with our results showing that the time after ON is inversely correlated with the total HRF count.

On the contrary, our data also reveal a positive correlation between neuroaxonal layer thickness (pRNFL, GCIPL) and HRF count in pwMS and pwNMOSD. Retinal neurodegeneration after ON is particularly characterized by apoptosis of retinal ganglion cells (RGC) in the GCIPL [34]. Histologic studies in EAE mice also showed that the early retrograde degeneration of RGC is mediated by microglia and enabled by the breakdown of the BRB [35, 36]. Yet, beyond the acute phase of ON when RGC loss already happened, BRB disruption and inflammation potentially decrease. As our cohort consisted predominantly of patients with ON in the chronic stage, a relevant GCIPL atrophy had already occurred. A histopathological study of post‐mortem retinal tissues of pwMS revealed an increased presence of retinal microglia in GCIPL and INL [37]. Further, retinal microglia were seen gathered around degenerated ganglion cells, particularly in the GCIPL [37]. While longitudinal studies will be necessary to fully understand the HRF dynamics, one could speculate that the formation of HRF is additionally dependent on the presence of RGCs as a substrate. This would also explain the higher numbers of HRF in ON^−^eyes compared to ON^+^eyes. Additionally, the dependence of HRF on RGC could also account for the missing association between HRF and INL thickness. INL thickening might reflect inflammatory activity in pwMS and is potentially caused by dynamic fluid shifts and Müller cell dysfunction [38] which are likely not primarily due to retinal microglia. However, due to the still not fully comprehended nature of HRF, all interpretations of the current results remain speculative. Further histopathological and in vivo examinations are therefore warranted.

CIS and early MS are considered to have similar pathologic mechanisms [1]. Both stages of the disease are characterized by active demyelinating lesions in the central nervous system with lymphocyte and microglia infiltrations [39]. After the 2017 iterations of the McDonald criteria, the amount of patients diagnosed with MS after a first demyelinating attack increased substantially [40]. As the diagnosis of CIS and MS comes closer, previous studies failed to find significant differences in immunological profiles between both diseases [41]. Therefore, we do not believe that differences in the number of HRF between CIS and MS are the result of alternating pathophysiologic mechanisms. Further, we ruled out an influence of age, sex, or disease duration as possible causes for differences in HRF numbers in pwCIS and pwMS. A potential explanation for the elevated number of HRF in pwCIS might be that the occurrence is influenced by immunomodulating medications. Even though none of the currently available disease‐modifying drugs for MS are directly targeting microglia, the indirect effects of these treatments are likely [42]. Additionally, the use of DMT was shown to slow down retinal neurodegeneration of INL and GCIPL in pwMS [43]. In our cohort, pwMS were only treated with low‐efficacy treatment due to the availability of treatments at the time of study inclusion and the short disease duration. Treated patients had numerically but not statistically fewer numbers of HRF compared to treatment‐naïve patients. Future prospective data will be necessary to confirm this effect. Further, the more pronounced retinal neurodegeneration in pwMS compared to pwCIS could be an additional confounder on HRF formation.

Strengths of the current study include the structured analysis of HRF using a software that was specifically developed for OCT evaluations, the exclusion of other concomitant eye diseases, and the correction of retinal segmentation by experienced raters. However, several limitations need to be discussed: We only performed a cross‐sectional analysis of OCT images. As HRF can change over time, longitudinal analysis might offer novel insights into the origin of HRF in neuroinflammatory diseases by evaluating possible changes in HRF numbers during relapses. Larger prospective trials are warranted to further assess the value of HRF as a prediction marker for future relapses. Also, OCT images of the acute phase of ON were not available. Experimental studies in EAE mice showed especially in the early stage of acute ON a pronounced increase in microglial activity [33]. Analysis of HRF in patients with acute ON might be an opportunity to reproduce these experimental results in humans. In addition, the comparability between pwNMOSD with pwCIS and pwMS is limited as pwNMOSD had a notably longer disease duration. Regarding pwMS, we only included patients with a relapsing–remitting disease course. As microglial activation is believed to be a key driver of clinical progression [42], future studies should evaluate HRF as a potential marker for progression in pwMS. Despite using a software specialized in the analysis of OCT images, an automated quantification of HRF would increase comparability and reduce the risk of human‐made errors. So far, no validated algorithm for automated segmentation of HRF is available even though some studies have attempted to establish one in diabetic retinopathy [44, 45]. Lastly, due to the structure of our data, blinding of the OCT images could only be done for ON status. We tried to reduce a potential rater bias by adhering to our protocol and ensuring quality control through analyzing by two separate raters. Still, in patients with severe ON or multiple previous ipsilateral ON, the raters could have detected a potential ON due to severe RNFL or GCIPL atrophy.

In conclusion, we demonstrated a higher number of HRF in INL and GCIPL in demyelinating diseases compared to HC. Due to the strong correlation of HRF to GCIPL thickness, HRF formation seems to be affected by RGC count. More advanced histopathologic studies are required to confirm that HRF represents activated microglia. Future clinical studies should also focus on establishing automated quantification software for HRF and subsequently evaluate the potential predictive utility of HRF, for example, for reconstitution of visual acuity after ON or disease activity independent of attacks.

## AUTHOR CONTRIBUTIONS

**Philipp Klyscz:** Conceptualization; methodology; writing – original draft; writing – review and editing; formal analysis; investigation; data curation; validation; visualization. **Ifat Vigiser:** Investigation; methodology; writing – review and editing. **Gilberto Solorza Buenrostro:** Software; writing – review and editing; data curation. **Seyedamirhosein Motamedi:** Writing – review and editing; software; data curation. **Carla Johanna Leutloff:** Writing – review and editing; data curation. **Patrick Schindler:** Writing – review and editing; investigation. **Tanja Schmitz‐Hübsch:** Funding acquisition; writing – review and editing; resources. **Friedemann Paul:** Resources; writing – review and editing; funding acquisition. **Hanna Gwendolyn Zimmermann:** Conceptualization; writing – review and editing; methodology. **Frederike Cosima Oertel:** Conceptualization; supervision; visualization; writing – review and editing; project administration; resources; funding acquisition.

## CONFLICT OF INTEREST STATEMENT

PKL, IV, GSB, SM, and CJL report no conflicts of interest. PS received travel support by UCB, received speaker‘s honoraria by Roche and Alexion, and served on an advisory board by Alexion. TSH reports speaker's honoraria from Honoraria AbbVie, Bayer, Roche and research grants bms/Celgene, and Roche. FP has received honoraria and research support from Alexion, Bayer, Biogen, Chugai, MerckSerono, Novartis, Genyzme, MedImmune, Shire, and Teva Pharmaceuticals, and serves on scientific advisory boards for Alexion, MedImmune, Novartis, and UCB. He has received funding from Deutsche Forschungsgemeinschaft (DFG Exc 257), Bundesministerium für Bildung und Forschung (Competence Network Multiple Sclerosis), Guthy‐Jackson Charitable Foundation, EU Framework Program 7, and National Multiple Sclerosis Society of the USA. He serves on the steering committee of the N‐Momentum study with inebilizumab (Horizon Therapeutics) and the OCTiMS Study (Novartis). He is an associate editor with Neurology, Neuroimmunology, and Neuroinflammation and academic editor with PloS One. HGZ reports grants and speaking honoraria from Novartis and personal fees from Bayer Healthcare, both unrelated to this project. FCO reports past research funding by the American Academy of Neurology, the National Multiple Sclerosis Society (US), and the German Association of Neurology (DGN). FCO reports current research support by the Hertie Foundation for Excellence in Clinical Neurosciences, by Novartis AG, by the DFG‐TWAS program—both unrelated to this project. She also reports speaker honoraria by UCB.

## Supporting information


Data S1.

## Data Availability

The datasets used and analyzed during the current study are available from the corresponding author on reasonable request.
