# Supplementary material for: Hyperreflective retinal foci are associated with retinal degeneration after optic neuritis in neuromyelitis optica spectrum disorders and multiple sclerosis
Source: Eur J Neurol. 2025 Jan 10;32(1):e70038. doi: 10.1111/ene.70038 (PMC11718220; doi:10.1111/ene.70038)
Supplement: Supplementary file 1 — Data S1. [file ENE-32-e70038-s001.pdf]

Supplementary Material

Hyperreflective retinal foci are associated with retinal degeneration after optic neuritis in neuromyelitis optica spectrum disorders and multiple sclerosis

Supplemental table 1. Uncorrected and corrected p values for correlations between number of HRF and retinal structural parameters in all eyes using false discovery rate correction.

	pwCIS		pwMS		pwNMOSD	
Correlations	uncorrected p value	corrected p value	uncorrected p value	corrected p value	uncorrected p value	corrected p value
Total HRF and pRNFL thickness	0.893	0.923	0.012	0.023	0.003	0.012
Total HRF and GCIPL thickness	0.686	0.762	0.026	0.046	0.010	0.020
Total HRF and INL thickness	0.519	0.655	0.299	0.407	0.999	0.999
HRF in GCIPL and GCIPL thickness	0.524	0.655	0.015	0.028	0.006	0.015
HRF in INL and INL thickness	0.255	0.364	0.844	0.904	0.612	0.706

Abbreviations: GCIPL, combined ganglion cell and inner plexiform layer; HRF, hyperreflective retinal foci; INL, inner nuclear layer; pRNFL, peripapillary retinal nerve fiber layer; pwCIS, people with clinically isolated syndrome; pwMS, people with multiple sclerosis; pwNMOSD, people with neuromyelitis optica spectrum disorder.

Supplemental table 2. Uncorrected and corrected p values for correlations between number of HRF and retinal structural parameters in ON^{+}eyes using false discovery rate correction.

	pwCIS		pwMS		pwNMOSD	
Correlations	uncorrected p value	corrected p value	uncorrected p value	corrected p value	uncorrected p value	corrected p value
Total HRF and pRNFL thickness	0.308	0.406	0.037	0.055	0.006	0.013
Total HRF and GCIPL thickness	0.386	0.421	0.024	0.043	0.041	0.067
Total HRF and INL thickness	0.722	0.744	0.620	0.713	0.105	0.174
HRF in GCIPL and GCIPL thickness	0.582	0.582	0.040	0.067	0.031	0.050
HRF in INL and INL thickness	0.612	0.713	0.825	0.825	0.116	0.182

Abbreviations: GCIPL, combined ganglion cell and inner plexiform layer; HRF, hyperreflective retinal foci; INL, inner nuclear layer; ON^{+}eyes, eyes with previous ON; pRNFL, peripapillary retinal nerve fiber layer; pwCIS, people with clinically isolated syndrome; pwMS, people with multiple sclerosis; pwNMOSD, people with neuromyelitis optica spectrum disorder.

Supplemental table 3. Uncorrected and corrected p values for correlations between number of HRF and retinal structural parameters in ON^{-}eyes using false discovery rate correction.

	pwCIS		pwMS		pwNMOSD	
Correlations	uncorrected p value	corrected p value	uncorrected p value	corrected p value	uncorrected p value	corrected p value
Total HRF and pRNFL thickness	0.054	0.138	0.324	0.562	0.525	0.713
Total HRF and GCIPL thickness	0.110	0.242	0.280	0.514	0.646	0.713
Total HRF and INL thickness	0.645	0.713	0.652	0.713	0.347	0.573
HRF in GCIPL and GCIPL thickness	0.216	0.420	0.374	0.588	0.503	0.713
HRF in INL and INL thickness	0.757	0.780	0.831	0.831	0.405	0.608

Abbreviations: GCIPL, combined ganglion cell and inner plexiform layer; HRF, hyperreflective retinal foci; INL, inner nuclear layer; ON^{-}eyes, eyes without previous ON; pRNFL, peripapillary retinal nerve fiber layer; pwCIS, people with clinically isolated syndrome; pwMS, people with multiple sclerosis; pwNMOSD, people with neuromyelitis optica spectrum disorder.
